# Editorial: Application of stem cells in degenerative orthopedic diseases

**DOI:** 10.3389/fcell.2026.1868045

**Published:** 2026-05-08

**Authors:** Jie Pan

**Affiliations:** Department of Pathology, Stanford University School of Medicine, Stanford, CA, United States

**Keywords:** bone-brain axis, extracellular vesicles, geroscience, mesenchymal stem cells, niche reprogramming

## 1 Introduction: the homeostatic shift

Degenerative orthopedic diseases, traditionally viewed as inevitable “wear-and-tear” processes, are now being reimagined as dynamic failures of tissue homeostasis orchestrated by a complex interplay of chronic inflammation and metabolic exhaustion. Over the past decade, the field of stem cell therapy, specifically involving mesenchymal stromal/stem cells (MSCs) and induced pluripotent stem cells (iPSCs) has transitioned from a simplistic “cell replacement” model to a sophisticated “niche reprogramming” paradigm. This Research Topic curates four pivotal contributions that integrate global clinical landscapes, bibliometric trajectories, and granular mechanistic insights to define the next-generation of regenerative orthopedics.

## 2 Mapping the translational landscape and evolving paradigms

A successful transition from bench to bedside requires resolving the structural paradoxes within clinical development and scientific focus. Li et al. provide an exhaustive mapping of 224 global clinical trials for osteoarthritis (OA), uncovering a “translational chasm” where the exponential expansion of early-phase trials stands in stark contrast to the scarcity of Phase III successes. This discrepancy reveals an urgent need for robust potency assays and precision-stratified patient cohorts to manage clinical heterogeneity.

Parallel to these clinical shifts, the bibliometric mapping by Ai et al. uncovers a decisive transition in the scientific center of gravity, where the focus has evolved from traditional structural scaffolds toward exosome-mediated biological signaling. This trajectory reflects a strategic industry-wide push toward cell-free paradigms, which circumvent the logistical and safety constraints of live-cell transplantation by utilizing stem cell-derived secretomes. Such systems offer superior scalability and refined immunogenic profiles, effectively redefining the standards for future regenerative products by prioritizing functional bio-signaling over mere structural replacement.

## 3 Mechanistic rigor: regenerating hostile pathological niches

Therapeutic efficacy in regenerative orthopedics is increasingly predicated on understanding the molecular cross-talk between therapeutic agents and their degenerate environment, particularly in complex systemic contexts. Xie et al. provide a compelling case for cell-free intervention by elucidating how chronic hyperglycemia in Diabetic Osteoarthritis biophysically rewires the MSC secretome. Their review underscores that MSC-derived exosomes (MSC-Exos) serve as critical mediators that can be engineered to counteract the NLRP3-mediated “pyroptotic” environment, emphasizing that future strategies must be comorbidity-aware to override systemic metabolic interference.

In a complementary yet fascinating mechanistic contrast, Junuzović et al. shift the focus back to the intricate physical interactions of cell-based therapies. By challenging the conventional paracrine-only dogma, they highlight the transformative role of mitochondrial transfer through tunneling nanotubes (TNTs). This suggests a dual-track regenerative logic: while cell-free exosomes offer a scalable, “off-the-shelf” approach to signaling modulation as discussed by Xie et al., the direct donation of functional organelles by live MSCs restores the bioenergetic integrity of exhausted chondrocytes at a foundational level. Together, these insights demonstrate that regenerating a hostile niche involves a sophisticated interplay between cell-free systemic signaling reprogramming and live-cell direct metabolic rescue, providing a multidimensional toolkit for personalized regenerative medicine.

## 4 Technological convergence: engineering the persistence of potency

The findings within this collection suggest that the “therapeutic ceiling” of regenerative orthopedics will be broken by the integration of bio-engineering and RNA biology. The development of injectable, responsive hydrogels and the molecular “loading” of specific microRNAs into extracellular vesicles are key strategies. These innovations aim to ensure that the regenerative signal persists long enough to override the proteolytic and pro-inflammatory environment characteristic of degenerative joints.

## 5 Future perspectives: the geroscience paradigm and the neuro-skeletal axis

As this Research Topic concludes, it is imperative to situate orthopedic regeneration within the broader framework of Geroscience, which posits that chronic degenerative diseases share a common set of biological drivers. The pathophysiology of musculoskeletal decay—traditionally viewed in isolation—is increasingly recognized as part of a systemic “geropathy” that mirrors the decline of the central nervous system (CNS) (Sui et al., 2022).

The “inflammaging” profile observed in the osteoarthritic joint—characterized by the accumulation of senescence-associated secretory phenotypes (SASP) (Rezuş et al., 2019; Diekman and Loeser, 2024; Han et al., 2024) and the failure of proteostatic surveillance—finds a profound parallel in the neurodegenerative milieu of Alzheimer’s and Parkinson’s diseases (Höhn et al., 2020; Pan et al., 2020). Both the articular chondrocyte and the post-mitotic neuron exist within highly specialized niches where the loss of cellular “tiling” and homeostatic polarization (e.g., the M1/M2 macrophage-microglia spectrum) leads to irreversible tissue loss (Zhang et al., 2025).

The emerging concept of the “Bone-Brain Axis” suggests a deep, bidirectional metabolic coupling between skeletal health and cognitive integrity (Zhang and Zhang, 2024; Yu J. et al., 2025). Osteokines and bone-derived extracellular vesicles (EVs) are no longer viewed as localized messengers but as systemic rheostats of inflammation (He et al., 2025). The engineered stem cell strategies discussed in this collection—designed to reprogram the joint microenvironment—hold transformative potential for neurobiology (Cheng et al., 2022). Specifically, the potential for MSC-derived EVs to cross the blood-brain barrier and modulate microglial proteostasis provides a unique opportunity to apply orthopedic breakthroughs to neuro-regeneration (Yu M. et al., 2025; Ye et al., 2023; Zheng et al., 2025).

Future research must pivot from organ-specific inquiries to a unified regenerative logic. Can the “mitochondrial rescue” mechanisms observed in mesenchymal-chondrocyte interactions be harnessed to restore bioenergetics in the aging neuro-glial unit? Could the anti-senescence RNAs refined for bone repair serve as systemic “senomorphic” agents to mitigate neuro-inflammation? By viewing orthopedic degeneration not as a localized mechanical failure but as a sentinel event in the systemic aging process, we can leverage these musculoskeletal innovations to tackle the multi-systemic decline that defines the human lifespan.

## 6 Conclusion

The contributions to this Research Topic demonstrate that regenerative orthopedics is entering a new era of mechanistic rigor. By moving from phenomenological observations to deep biological and translational insights, we are paving the way for therapies that do not merely mask symptoms but actively restore the vitality of the human musculoskeletal system—and potentially, the systemic health of the aging population.
